# Impaired Windkessel function and proximal aortic stiffness: Linking vascular ageing to cognitive decline

**DOI:** 10.1113/EP092672

**Published:** 2025-12-18

**Authors:** Jun Sugawara, Hirofumi Tanaka

**Affiliations:** ^1^ Human Informatics and Interaction Research Institute National Institute of Advanced Industrial Science and Technology (AIST) Tsukuba Japan; ^2^ Faculty of Health and Sport Science University of Tsukuba Tsukuba Japan; ^3^ Department of Kinesiology and Health Education The University of Texas at Austin Austin Texas USA; ^4^ Faculty of Sport Science Chulalongkorn University Bangkok Thailand

**Keywords:** ageing, arterial stiffness, cerebrovascular disease, dementia, physical activity

## Abstract

Central arterial stiffening, particularly of the proximal aorta, is increasingly recognised as a pivotal contributor to cardiovascular disease, dementia, and mild cognitive impairment. Loss of Windkessel function amplifies pulsatile pressure, reduces diastolic perfusion and accelerates microvascular damage in the brain. Evidence from epidemiological studies, magnetic resonance imaging investigations and longitudinal data demonstrates disproportionate age‐related stiffening of the proximal aorta and its strong association with cognitive decline. Importantly, this process is modifiable: aerobic exercise and unique environmental adaptations, such as those observed in Japanese Ama divers, preserve proximal aortic elasticity. Targeting central arterial stiffness may represent a promising strategy for preventing both vascular disease and brain dysfunction.

## Abstract (highlights)

- **What is the topic of this review?**
  This review focuses on central arterial stiffening, especially of the proximal aorta, as a key link between vascular ageing, cardiovascular disease and cognitive decline. Loss of Windkessel function amplifies pulsatile pressure, reduces diastolic perfusion and accelerates microvascular injury, which would be risks for cognitive decline.
- **What advances does it highlight?**
  This review integrates emerging evidence that both structured aerobic training and lifelong environmental activities, such as breath‐hold diving in Japanese Ama, maintain proximal aortic compliance and cushioning capacity. These adaptations attenuate pulsatile energy transmission to the brain, preserve cerebral perfusion, and may mitigate age‐related cognitive decline. Thus, maintaining Windkessel function through physical and environmental adaptation represents a promising strategy to counteract vascular and brain ageing.

## 1 INTRODUCTION

Cardiovascular disease (CVD) has remained the leading cause of morbidity and mortality for decades, representing a persistent global health challenge (Amini et al., 2021; Di Cesare et al., 2024). In parallel, the prevalence of dementia and its prodromal stage, mild cognitive impairment (MCI), continues to rise as populations age (Prince et al., 2015; WHO, 2017). These two major public health concerns are often interconnected through shared vascular risk factors and pathophysiological mechanisms. Indeed, vascular dysfunction appears to be the earliest event that leads to dementia and Alzheimer's disease (AD), showing up well before metabolic, functional, and structural brain declines (Iturria‐Medina et al., 2016). In particular, a growing body of evidence indicates that age‐related increases in central arterial stiffness are a key contributor to the development and progression of both cardiovascular and neurocognitive disorders (Liu et al., 2021; Meyer et al., 2017; van Sloten et al., 2015; Vlachopoulos et al., 2010). With increasing arterial stiffness, the resulting haemodynamic changes, including heightened pulsatile pressure and impaired pulsatile pressure buffering, can damage both the heart and the brain. Most previous studies have assessed arterial stiffness indirectly using pulse wave velocity (PWV), which primarily reflects regional arterial stiffness as it propagates along the arterial tree rather than as buffering or cushioning of pulsations (Tanaka, 2017). Additionally, these measures generally exclude the proximal aortic segment connecting the heart and the brain, the key site responsible for Windkessel function and pulsatile flow cushioning.

This brief review seeks to synthesise current understanding of the mechanisms by which arterial stiffening contributes to cardiovascular and cognitive decline, paying particular attention to the buffering and cushioning capacity of the proximal aorta, known as the Windkessel function. Furthermore, we highlight how habitual physical activity and exercise can induce favourable adaptations in central arterial function. By clarifying these physiological mechanisms and adaptations, this review aims to inform preventive strategies that target vascular ageing as a means of reducing the burden of both CVD and dementia.

## 2 WINDKESSEL FUNCTION IN THE PROXIMAL AORTA

A German physiologist, Otto Frank, coined the term *Windkessel* (air chamber) when he observed that the cushioning function of the aorta resembles the air reservoir of a fire engine, both acting to buffer the pulsatile output of an intermittent pump into a steady flow downstream (Frank, 1899) (Figure 1). Compared with the peripheral conduit arteries in the limbs, the aorta and carotid arteries – collectively referred to as central arteries – contain a much higher proportion of elastin within their walls. This elastin‐rich structure allows them to expand during systole and recoil during diastole, thereby buffering the pulsatile output of the heart and maintaining continuous flow to the periphery and coronary circulation, effectively acting as an auxiliary pump during diastole (Belz, 1995). Frank later formalised these mechanical principles in his two‐element *Windkessel* model, which describes the elastic and resistive properties of the arterial system (Frank, 1899; Parker, 2009; Sagawa et al., 1990).

**FIGURE 1:**
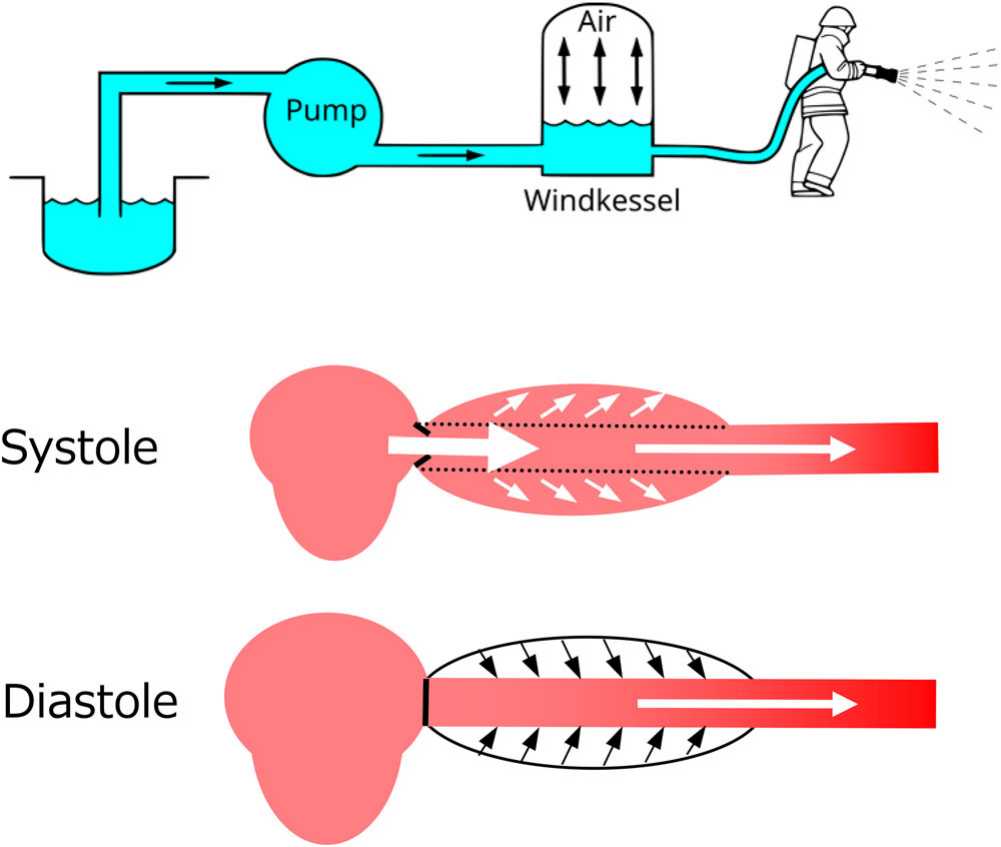
Conceptual illustration of the aortic Windkessel function. During systole, the elastic aorta expands to store part of the ejected stroke volume as potential energy, and during diastole, it recoils to sustain blood flow and stabilise pressure when the heart is not ejecting. Otto Frank likened this buffering effect to the air reservoir in a fire engine pump that smooths intermittent piston strokes into continuous outflow. Likewise, the aortic Windkessel converts the pulsatile cardiac output into a more steady flow, reducing excessive pressure transmission to downstream organs such as the brain and kidneys. Modified in part from 'Windkessel effect' by Kurzon, Wikimedia Commons (https://commons.wikimedia.org/wiki/File:Windkessel_effect.svg.

However, with advancing age, the distensibility of central arteries declines due to a variety of factors, including elastin fragmentation, collagen accumulation, medial thickening, and calcification (Lakatta & Levy, 2003). In addition to these structural changes, age‐related functional changes such as heightened sympathetic nerve activity and endothelial dysfunction contribute to increased arterial stiffness (Holwerda et al., 2019; Seals et al., 2011; Tanaka et al., 2017). Stiffening of central elastic arteries impairs Windkessel function and places a substantial burden on the heart, presumably due to augmented afterload and diminished coronary perfusion. Based on the accumulated evidence from early cohort studies (Blacher et al., 1999; Boutouyrie et al., 2002; Laurent et al., 2001; Meaume et al., 2001; Shoji et al., 2001), central arterial stiffening has been established as a predictor of CVD.

## 3 WINDKESSEL FUNCTION IN PROTECTING THE BRAIN

Beyond its effects on the heart, the Windkessel function of the central arteries also plays a crucial role in maintaining cerebral circulation and brain health. Organs such as the brain and kidneys are characterised as high‐flow, low‐resistance systems because their resistance vessels are relatively well‐dilated to allow a continuous supply of blood to support their high metabolic demands and specialised functions (Mitchell, 2018). Because these organs lack extensive resistance upstream, they are directly exposed to fluctuations in systemic blood pressure and flow, imposing mechanical stress that leads to structural damage (Palta et al., 2019). Under normal conditions, as shown in Figure 2a, the Windkessel function of the central arteries plays a critical protective role against such injury (Palta et al., 2019). However, with increasing arterial stiffness, the cushioning capacity is diminished, resulting in the direct transmission of pulsatile stress into fragile microvascular beds of target organs such as the brain (Mitchell, 2018; O'Rourke & Hashimoto, 2007; O'Rourke & Safar, 2005) (Figure 2b).

**FIGURE 2:**
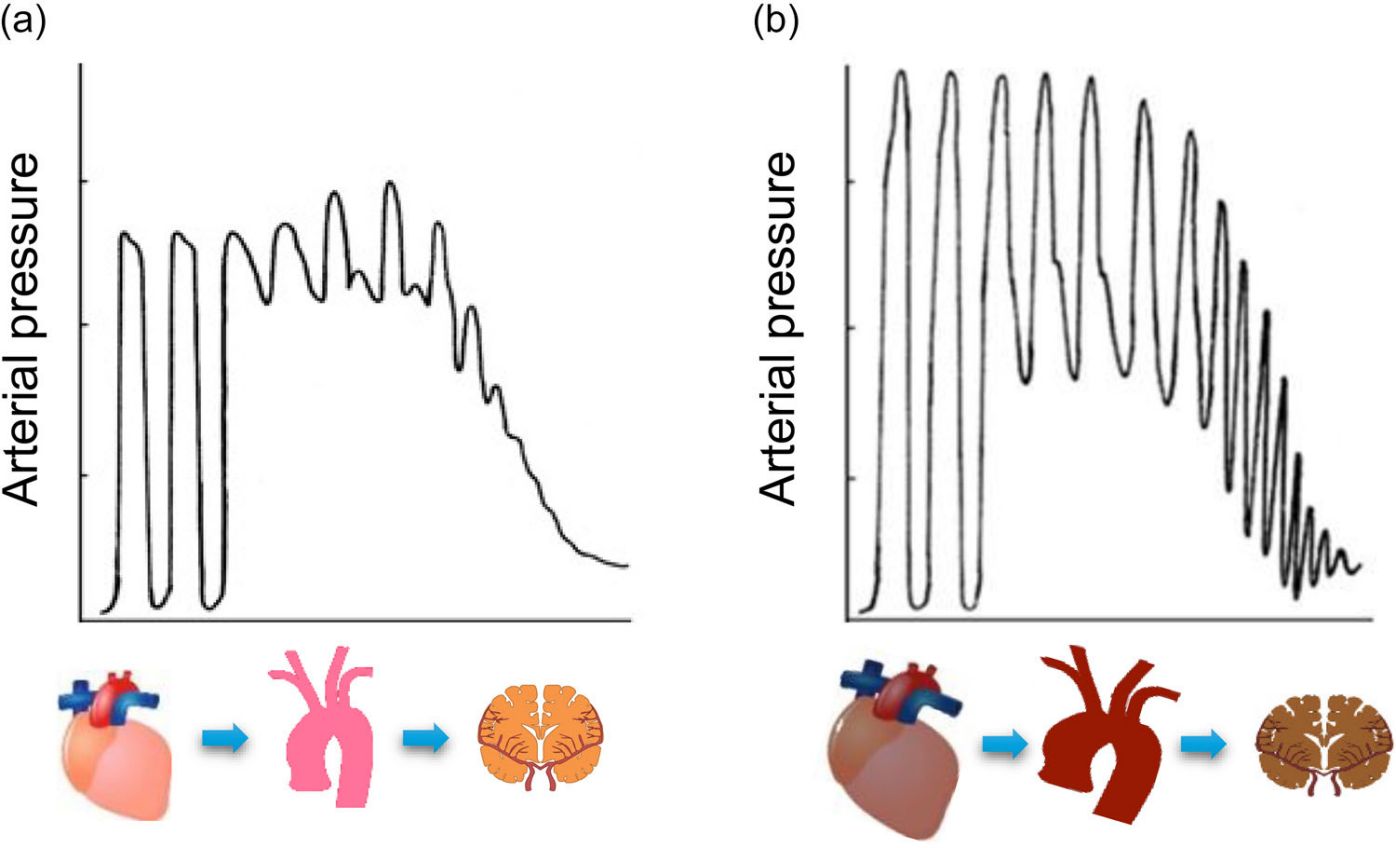
Impact of Windkessel function on pulsatile haemodynamics. Illustration of preserved (a) and impaired (b) aortic Windkessel function. When the Windkessel mechanism is preserved, the elastic aorta buffers pulsatile ejection from the heart, reducing systolic pressure, and maintaining diastolic flow. In contrast, with impaired elasticity, this buffering capacity is diminished, leading to augmented pressure pulsatility and increased load on downstream organs. Adapted and modified from O'Rourke & Hashimoto (2007).

Arterial stiffening with advancing age is substantially greater in central elastic arteries than in peripheral muscular arteries (Avolio et al., 1983; Sugawara et al., 2019), with the proximal aorta most affected. In adults younger than 55 years, arterial stiffness as measured by magnetic resonance imaging (MRI) is relatively uniform across aortic segments, but after midlife, the proximal aorta stiffens markedly compared with distal regions (Redheuil et al., 2010). A cross‐sectional observation using 4D flow MRI indicates that ascending aortic PWV increases by ∼16% per decade (Jarvis et al., 2022). Collectively, these data highlight that proximal aortic stiffening is pronounced with increasing age and plays a pivotal role in age‐related haemodynamic burden, particularly by compromising the cushioning of pulsatile flow and protection of the cerebral circulation.

Each ventricular contraction imposes mechanical stress on target organs repeatedly over a long period of years. The heart contracts and relaxes continuously with more than 100,000 contractions per day at a constant heart rate of 70 bpm at rest. This phenomenon of repeated stress illustrates the ancient maxim *Gutta cavat lapidem* (Ovid, *Epistulae ex Ponto*), ‘a drop hollows a stone’, or in a longer version, ‘a drop of water hollows a stone, not through force but through persistence’. The observation that central arterial stiffness increases the risk of target‐organ damage, such as cerebrovascular disorders and kidney disease, can be understood in the context of this physiological mechanism.

Indeed, emerging evidence has highlighted the role of central arterial stiffness in cerebrovascular pathology. A systematic review and meta‐analysis integrating 23 studies on cerebral small vessel disease and 41 studies on cognition demonstrated that greater arterial stiffness, assessed by a variety of techniques, including carotid‐femoral PWV (cfPWV) and brachial‐ankle PWV (baPWV), carotid artery compliance or pulse pressure, is consistently associated with an increased risk of cerebral small vessel disease (van Sloten et al., 2015). However, findings for cognitive impairment were more heterogeneous, and the overall associations between arterial stiffness and cognition are modest (van Sloten et al., 2015). Subsequent work (Liu et al., 2021) has begun to clarify these relations. A recent systematic review and meta‐analysis synthesising 39 studies qualitatively and 29 studies quantitatively reported that greater aortic PWV was significantly associated with poorer memory and slower processing speed (Liu et al., 2021). Longitudinal analyses further showed that individuals with higher PWV had a 44% greater risk of cognitive impairment, with each 1 m/s increase in PWV conferring an additional 3.9% risk (Liu et al., 2021). Notably, these associations are particularly evident in older adults, suggesting that aortic stiffness, as assessed by PWV, may serve as an independent risk marker of cognitive decline with advancing age.

## 4 MECHANISMS LINKING ARTERIAL STIFFNESS TO COGNITIVE DECLINE

Brain hypoperfusion is a well‐recognised feature of AD and MCI, the prodromal stage of AD (Duncombe et al., 2017; Iadecola, 2004; Liu et al., 2014). Both global and regional reductions in cerebral blood flow (CBF) have been attributed to cardiovascular characteristics such as reduced cardiac output, hypertension, endothelial dysfunction, and atherosclerosis, as well as to brain features including cerebral hypometabolism, impaired neuronal protein synthesis, and disrupted neurovascular coupling (Duncombe et al., 2017; Leeuwis et al., 2017; Tarumi & Zhang, 2018). Elevated cerebral vasoconstriction may further aggravate CBF reduction in AD and MCI (Nation et al., 2013; Tomoto et al., 2020). Importantly, amyloid‐β (Aβ), a pathological hallmark of AD, can induce cerebral vasoconstriction via α_1_‐adrenergic receptors (Haase et al., 2013), thereby impairing CBF regulation and worsening brain hypoperfusion.

Recent evidence links central arterial stiffening to these cerebrovascular changes. In cognitively normal adults varying widely in age, carotid β‐stiffness index and cfPWV were inversely related to cerebral perfusion (Ashley et al., 2025). Specifically, age was negatively associated with total CBF, whilst internal carotid artery diastolic velocity was inversely correlated with both cfPWV and carotid β‐stiffness. These findings suggest that increased central arterial stiffness, through impaired Windkessel function – particularly reduced diastolic recoil – may lower diastolic velocity in the internal carotid artery, thereby contributing to age‐related declines in CBF. An alternative hypothesis is that a failure to control perfusion pressure due to impaired baroreflex sensitivity, a well‐known sequelae of arterial stiffening, could potentially contribute to chronic brain hypoperfusion, leading to cognitive dysfunction (Laosiripisan et al., 2015).

Converging evidence in patients with MCI (Tomoto et al., 2020) further supports this link. Individuals with amnestic MCI exhibit lower normalised CBF and higher cerebrovascular resistance compared with age‐ and sex‐matched controls. Carotid β‐stiffness index was independently associated with reduced CBF, whilst carotid systolic pressure predicted higher cerebrovascular resistance, implicating carotid arterial stiffening as a key determinant of impaired cerebrovascular haemodynamics in MCI. Extending these observations, a study in older adults with amnestic MCI (Pasha et al., 2020) showed that carotid β‐stiffness index correlated positively, and carotid distensibility negatively, with cortical and precuneus Aβ deposition, implicating stiffening along the arterial pathway from the heart–brain vascular axis in AD pathology.

Beyond these empirical findings, a compensatory yet maladaptive vascular mechanism has been proposed. In response to increased pulsatile stress from central arterial stiffening, the cerebral vasculature may elevate vascular smooth muscle tone to shield microcirculation from structural damage (Sugawara et al., 2021, 2022). However, this response may also act to increase cerebrovascular impedance and reduce cerebral perfusion. Indeed, cerebrovascular impedance increases with advancing age and correlates with indices of arterial stiffness (Sugawara et al., 2021). Similar patterns in MCI patients suggest that arterial stiffening may accelerate cognitive decline not only through hypoperfusion and amyloid accumulation but also via maladaptive vascular protective responses.

In parallel, the glymphatic system, which facilitates cerebrospinal fluid movement along periarterial spaces (Iliff et al., 2012; Jessen et al., 2015), has gained recognition as a key pathway for metabolic waste clearance. Animal studies provide critical insights: vessel wall distension of cerebral arterioles declines with ageing (Kress et al., 2014) and is likewise reduced in transgenic AD models (Li et al., 2021). This is associated with impaired Aβ clearance (Li et al., 2021). These findings suggest that diminished vessel wall pulsatility with ageing or disease compromises perivascular clearance. Taken together, these findings position arterial stiffness as a risk factor for the onset and progression of AD, integrating haemodynamic compromise, amyloid pathology and maladaptive vascular responses into a unifying pathophysiological framework.

## 5 HABITUAL EXERCISE AND CENTRAL ARTERY STIFFNESS

In contrast to the notion that arterial stiffness cannot be changed because it is determined by seemingly rigid arterial structures, central arterial stiffness is highly modifiable through lifestyle interventions, most notably via habitual exercise (Seals, 2003; Seals et al., 2009; Tanaka & Safar, 2005). Indeed, numerous studies have reported beneficial effects of regular exercise on central and systemic arterial stiffness as measured by cfPWV and baPWV (Fujie et al., 2020; Hasegawa et al., 2018; Kakiyama et al., 2005; Sugawara et al., 2005; Sugawara, Tomoto, Noda, et al., 2018; Zempo‐Miyaki et al., 2016). It should be noted that both cfPWV and baPWV rely on pulse wave foot detection on two sites (carotid or brachial arteries) and do not include the segment between the aortic root and the branching points of the carotid and brachiocephalic arteries. Therefore, due to methodological limitations, cfPWV and baPWV do not directly capture the stiffness of the proximal aorta, the primary site for the Windkessel function. However, research studies using carotid artery stiffness and compliance measured by combined ultrasonography and applanation tonometry (Moreau et al., 2003; Shibata et al., 2018; Sugawara et al., 2006; Tanaka et al., 2000; Tomoto et al., 2021, 2015) have demonstrated the benefits of habitual exercise on central artery stiffness.

Recent advances in MRI have enabled detailed assessment of proximal aortic structure and function. A series of studies employing phase‐contrast and cine‐mode MRI with simultaneous blood pressure and cardiac measurements compared young male endurance athletes with sedentary controls (Fukuie et al., 2024; Tarumi et al., 2021). Stroke volume was greater, and ascending and descending aortas were larger and more compliant in athletes. Additionally, ascending aortic compliance was positively related to stroke volume (Tarumi et al., 2021). Moreover, characteristic impedance (ZcF) was lower in athletes, reflecting reduced pulsatile afterload. This is noteworthy as ZcF emerged as the strongest predictor of stroke volume, underscoring its role in ventricular–vascular coupling (Fukuie et al., 2024). Collectively, these findings highlight enhanced proximal aortic compliance and reduced afterload as key vascular adaptations to endurance training.

## 6 ENVIRONMENTAL ADAPTATIONS OF THE PROXIMAL AORTA

Intriguing evidence has emerged regarding the proximal aortic adaptations resulting from the synergistic interaction of environmental factors and habitual physical activity (Sugawara, Tomoto, Lin, et al., 2018; Tanaka et al., 2016). The Ama are Japanese female pearl divers who practice breath‐hold free diving in the ocean, with origins referenced as early as 268 BC in the *Gishi‐Wajin‐Den*, one of the earliest written descriptions of Japan and its people in a Chinese historical record. It is historically believed that women are better suited for diving due to greater cold tolerance and fat insulation. Ama continues to dive without modern equipment to protect ecosystems and reduce overfishing. Even today, they perform ∼100–150 dives per day, each lasting up to 2 min. The diving bradycardia is quite pronounced, with heart rate falling to nearly 50% of baseline values (Scholander et al., 1962), and is further exaggerated during cold water immersion in the wintertime (Ferrigno et al., 1997). Cold water immersion also provokes a marked rise in arterial blood pressure (Ferrigno et al., 1997; Kawakami et al., 1967). To counteract this circulatory stress, the proximal aorta plays a key role in reducing cardiac workload through its Windkessel function – storing blood by arterial wall distension during systole and sustaining diastolic pressure and coronary perfusion via elastic recoil throughout the prolonged diastolic phase. Indeed, a simulated free diving manoeuvre that combines exercise, apnoea, and facial immersion in the laboratory can reduce arterial stiffness to help buffer cardiac pulsations (Fico et al., 2022). Moreover, diving mammals (e.g., fin whales) demonstrate a well‐adapted arterial structure that is mechanically very different from other mammals, as the elastic modulus of the aorta is markedly greater than other mammals, and the aortic arch is substantially enlarged relative to the thoracic aorta (Shadwick & Gosline, 1995).

Lifelong female Ama divers demonstrate significantly lower arterial stiffness as measured by cardio–ankle vascular index than age‐matched women from the same fishing village, corresponding to a vascular age that was, on average, 11 years younger (Tanaka et al., 2016). Furthermore, they exhibit superior proximal aortic reservoir function relative to age‐matched sedentary women (Sugawara, Tomoto, Lin, et al., 2018), as evidenced by lower reservoir and excess‐pressure integrals and a reduced characteristic impedance, which likely reflect more compliant proximal aortic properties. Although the ratio of the excess‐pressure integral to the reflected‐pressure integral did not differ between groups, the lower absolute integrals in Ama likely reflect their overall lower pressure levels and attenuated wave‐related pulsatile components, consistent with more favourable proximal aortic cushioning function. Notably, the occupational activity of Ama diving does not qualify as conventional aerobic exercise, which can be maintained continuously and is rhythmic in nature, and is widely recognised as effective in improving arterial stiffness. Instead, it involves repeated bouts of breath‐hold diving followed by rests above the water.

Collectively, these studies provide novel insights into adaptations of arterial Windkessel function induced by unique forms of physical activity and environmental exposure (Fukuie et al., 2021; Sugawara, Tomoto, Lin, et al., 2018; Tanaka et al., 2016). Whether Ama divers exhibit a lower incidence of dementia compared with the general female population remains unclear, highlighting the need for further investigation.

## 7 IMPROVED WINDKESSEL FUNCTION, CEREBRAL HAEMODYNAMICS AND COGNITIVE FUNCTION

A systematic review and meta‐analysis found that higher cardiorespiratory fitness is associated with greater middle cerebral artery (MCA) blood velocity in older adults based on cross‐sectional studies (Smith et al., 2021). However, intervention studies of moderate‐intensity aerobic exercise training for 2–12 months showed little effect on MCA velocity or global cerebral perfusion measured by MRI‐arterial spin labelling, suggesting that the effects of aerobic exercise training on CBF remain inconclusive (Smith et al., 2021). However, a more recent systematic review and meta‐analysis concluded that exercise enhances cognitive function in older adults, partly by improving macrovascular CBF and cardiovascular efficiency (Li et al., 2025). In one of the intervention studies included, 1 year of aerobic exercise training increased global CBF, reduced cerebrovascular resistance and decreased carotid arterial stiffness in both cognitively normal older adults (Tomoto et al., 2023) and those with MCI (Tomoto et al., 2021). These adaptations were largely mediated by increased internal carotid artery flow, and reductions in carotid stiffness underpin the associations between improved cardiorespiratory fitness and favourable cerebrovascular outcomes. In cognitively normal older adults, cerebrovascular impedance was also reduced after a 1‐year aerobic exercise intervention, which may benefit brain perfusion (Sugawara et al., 2022). However, in the study involving MCI patients, no significant improvement in cognitive function has been reported following moderate walking exercise lasting 1 year (Tomoto et al., 2021). Conversely, Nordic walking exercise in water is a novel whole‐body low‐impact exercise that could activate greater parts of the motor cortex and elevate cerebral perfusion via hydrostatic pressure. Aquatic Nordic walking improved vascular function (e.g., carotid arterial compliance, baPWV, and flow‐mediated vasodilation), cerebrovascular reactivity and cognitive performance in older adults with MCI and type 2 diabetes, with positive correlations between improvements in cerebrovascular reactivity and cognition (Ploydang et al., 2023).

## 8 CONCLUSION

Central arterial stiffness – especially within the proximal aorta – emerges as a pivotal determinant of cardiovascular, cerebrovascular, and cognitive health. Loss of Windkessel function amplifies pulsatile stress, diminishes organ perfusion, and accelerates end‐organ damage. Yet, proximal aortic stiffness is not an inexorable consequence of ageing. It is modifiable through habitual exercise and, intriguingly, through unique environmental activities such as free diving and aquatic Nordic walking. Preserving central arterial elasticity, and particularly the Windkessel function, may therefore represent an effective preventive strategy for dementia and related age‐associated diseases.

## AUTHOR CONTRIBUTIONS

Drafting the manuscript: Jun Sugawara. *Providing critical revisions*: Hirofumi Tanaka. All authors contributed to the conception and design of the study. Both authors have read and approved the final version of this manuscript and agree to be accountable for all aspects of the work in ensuring that questions related to the accuracy or integrity of any part of the work are appropriately investigated and resolved. All persons designated as authors qualify for authorship, and all those who qualify for authorship are listed.

## CONFLICT OF INTEREST

The authors declare no potential conflicts of interest with respect to the research, authorship and/or publication of this article.
